# Complete Genomic Sequence of Southern Rice Blacked-Dwarf Virus, a Novel Fijivirus, from Vietnam

**DOI:** 10.1128/genomeA.00212-13

**Published:** 2013-05-23

**Authors:** Jin Xue, Jing Li, Hoang-Anh Ta, Heng-Mu Zhang, Jian Yang, Ming-Fang Lv, Yuan Meng, Pei-Pei Li, Jian-Ping Chen

**Affiliations:** College of Bio-Safety Science and Technology, Hunan Agricultural University, Changsha, Chinaa; State Key Laboratory Breeding Base for Zhejiang Sustainable Pest and Disease Control, Key Laboratory of Plant Protection and Biotechnology, Ministry of Agriculture, and Zhejiang Provincial Key Laboratory of Plant Virology, Institute of Virology and Biotechnology, Zhejiang Academy of Agricultural Sciences, Hangzhou, Chinab; Plant Protection Research Institute, Hanoi, Vietnamc; College of Agriculture and Biotechnology (CAB), Zhejiang University, Hangzhou, Chinad

## Abstract

The nucleotide sequences of the ten genomic segments of a Vietnam isolate of southern rice blacked-dwarf virus were determined. This complete genomic sequence will help to further understand the viral etiology (origin of viral pathogen) and phylogenetic relationships among fijiviruses.

## GENOME ANNOUNCEMENT

Southern rice black-streaked dwarf virus (SRBSDV) (or rice black-streaked dwarf virus 2) was first reported in China and was identified as a novel putative member of the genus *Fijivirus* within the family *Reoviridae* (1–5). SRBSDV has morphological and serological similarities to rice black-streaked dwarf virus (RBSDV), a recognized member of the genus *Fijivirus*, but it is efficiently transmitted by the white-backed planthopper (*Sogatella furcimera* Horváth) (3, 6) and only poorly transmitted by the small planthopper (*Laodelphax striatellus* Fallen), the main vector of RBSDV (4, 5).

In Vietnam, a novel dwarf and leaf-twisting syndrome was first observed on rice plants in Nghe An Province in the summer 2009 crop. The disease rapidly spread among diverse agroecological regions in north and central Vietnam and also infected maize after rice was harvested. Although the disease was at first thought to be caused by a mixed infection of rice grassy stunt virus (RGSV) and rice ragged stunt virus (RRSV), transmission experiments and partial genomic data allowed us to identify the causal agent as SRBSDV (7). To date, SRBSDV has been reported from at least 35 provinces and cities in Vietnam (8). The complete sequences of all 10 double-stranded RNA (dsRNA) genome segments of two SRBSDV isolates (SRBSDV-GD and SRBSDV-HN) from China have been determined (9). However, only partial sequences of Vietnamese isolates have been reported (7). To understand the etiology of the viral disease in Vietnam and the phylogenetic relationships among fijiviruses, we have therefore determined the complete genomic sequence of a Vietnam isolate of SRBSDV (SRBSDV-V). Viral dsRNA purification, ligation-dependent RT-PCR amplification, and cloning were performed as described previously (10, 11). The inserts were sequenced using the BigDye Terminator v3.1 cycle sequencing kit on an ABI Prism 3730 DNA sequencer (PerkinElmer Applied Biosystems). The sequences were assembled using the DNAman v6.0 program (Lynnon Corporation, Canada).

The total ten-segment genome of SRBSDV-V had 29,115 nucleotides (nt), which is 9 nt shorter than SRBSDV-GD or SRBSDV-HN but similar in organization to these two Chinese isolates and other group 2 fijiviruses that have been sequenced (9–13). Each of the genome segments S1 to S4, S6 and S8, and S10 encodes a single major protein, while S5, S7, and S9 each have two nonoverlapping or partially overlapping open reading frames (ORFs). The extreme 5′ and 3′ ends of the sense strand of each segment have the sequence 5′-AAGTTTTT. …CAGCTA(G)T(C/A)T(C)GTC-3′, which is similar to the conserved terminal sequences reported from RBSDV, Mal de Río Cuarto virus (MRCV), and maize rough dwarf virus (MRDV) segments (9–13). The 13 ORFs share the highest homologies (96 to 99%) with those of the corresponding segments of SRBSDV and share some homologies to those of other fijiviruses. In the phylogenetic analyses of their segments, SRBSDV, RBSDV, MRDV, and MRCV were always closely clustered together, supporting the hypothesis that SRBSDV should be a novel species in group 2 of the genus *Fijivirus*, in the family *Reoviridae* (1, 3). The new genomic sequences of SRBSDV-V will be important for determining the taxonomic position of the isolate and the etiology of the viral disease in Vietnam.

### Nucleotide sequence accession numbers.

The complete genomic sequence of SRBSDV-V has been deposited in the GenBank/EMBL/DDBJ databases under the accession no. HQ731492 to HQ731501, corresponding to genomic segments 1 through 10, respectively.
